# Influence of Non-conventional Sperm Quality Parameters on Field Fertility in Ovine

**DOI:** 10.3389/fvets.2021.650572

**Published:** 2021-05-07

**Authors:** Noelia Mendoza, Adriana Casao, Juan Domingo, Francisco Quintín, Adolfo Laviña, Enrique Fantova, José Álvaro Cebrián-Pérez, Teresa Muiño-Blanco, Rosaura Pérez-Pe

**Affiliations:** ^1^Grupo BIOFITER-Departamento de Bioquímica y Biología Molecular y Celular—Instituto Universitario de Investigación en Ciencias Ambientales de Aragón (IUCA), Facultad de Veterinaria, Universidad de Zaragoza, Zaragoza, Spain; ^2^Centro de Transferencia Agroalimentaria (Gobierno de Aragón), Zaragoza, Spain; ^3^Asociación nacional de criadores de ganado ovino de la raza rasa aragonesa (ANGRA), Zaragoza, Spain; ^4^Unión de Productores de Raza Rasa Aragonesa (UPRA)-Grupo Pastores, Zaragoza, Spain

**Keywords:** ram, semen, quality, season, fertility, capacitation, apoptosis

## Abstract

The prediction of the fertilizing ability of a seminal dose continues to be a primary aim in the field of artificial insemination (AI). To achieve this goal, in this study we have included the evaluation of some non-conventional sperm quality markers. A total of 3,906 ewes from 52 different farms were inseminated with 357 refrigerated seminal doses obtained from 45 mature *Rasa Aragonesa* rams. The same samples were used for sperm quality analysis including membrane integrity, capacitation status, oxygen consumption and apoptotic-like markers such as phosphatidylserine translocation (PS), plasmalemma disorganization/mitochondrial membrane potential, caspase activation and DNA damage. Seminal doses from the breeding (B) season presented higher percentages of intact membrane (IM), non permeant (NP) membrane with high mitochondrial membrane potential (ΔΨm) and IM without PS translocation spermatozoa than those from the non-breeding (NB) season. Therefore, we can conclude that there were less spermatozoa showing apoptotic-like features in the seminal doses from the B than the NB season, although these differences did not affect field fertility. Only the percentage of intact membrane, non-capacitated (IM-NC) spermatozoa showed a significant correlation with *in vivo* fertility (*P* = 0.005) and fecundity (*P* = 0.007) values obtained after cervical AI when all data were evaluated. When the data were sorted by season and distance to the farms where AI was performed, the correlation between the percentage of IM-NC spermatozoa and reproductive parameters increased in the NB season and progressively with remoteness from the farms. Some other sperm parameters, like NP with high ΔΨm, IM sperm without active caspases and DNA-intact spermatozoa, also showed significant correlations with the reproductive parameters in the sorted data. Moreover, the increment in both the percentage of IM-NC and DNA-intact spermatozoa would increase the probability of obtaining a fertility higher than the mean (>52%), as revealed by a multiple logistic regression analysis. In conclusion, we have identified two seminal markers—the percentage of intact membrane, non-capacitated spermatozoa, and DNA intact spermatozoa—which could be used as a test to discard males in AI programs, which is highly important from an economic point of view and can contribute to achieving satisfactory fertility rates.

## Introduction

The use of spermatozoa with high fertilizing potential is critical for obtaining good results in reproductive technologies with domestic animals, but this implies the need to know in advance the fertilizing ability of a seminal dose. It is generally assumed that ejaculated spermatozoa with good fertilizing capacity must have progressive motility, normal morphology and intact plasma and acrosomal membranes. However, the standard semen analyses based only on these parameters do not permit an accurate assessment of all sperm functional characteristics, due to over- or under-estimating the fertilizing capacity of a given sample (1–3). Therefore, the consideration of new aspects for the assessment of sperm functionality might help to establish the quality of a seminal dose (4, 5). Fertilization is a process that requires several sperm capacities, and thus the combination of different analysis techniques of semen quality and/or sperm functionality would allow improving the predictive power for the fertility rate (6–8).

Capacitation is a prerequisite for achieving fertilization, but this sperm state must be attained in the oocyte's surroundings. It is well-known that cryopreservation induces an increment in the percentage of capacitated spermatozoa but, in certain species, this increment happens even during cooling to temperatures above freezing (9–12). So, this premature capacitation could diminish the fertility achieved with refrigerated seminal doses when artificial insemination is carried out. Refrigeration has also accounted for an increase in spermatozoa showing certain features related with apoptosis in somatic cells, such as phosphatidylserine translocation, DNA fragmentation, activation of caspases or mitochondrial impairment (13–15). All these types of sub-lethal damage could also impair the reproductive success of refrigerated seminal samples. However, the detrimental effect of apoptotic spermatozoa on field fertility in ovine has not been reported to date. Oxygen is essential for the generation of cellular energy (ATP) via oxidative phosphorylation, and thus oxygen consumption is a key indicator of metabolic activity within cells (16). However, there are scarce reports that link oxygen consumption and fertility (17).

Fertility results in ovine are influenced by seasonality. Seasonal changes in testicular size, sperm production, mating activity and fertility have been described in rams (18, 19). Likewise, differences between breeding and non-breeding seasons in some sperm quality parameters and seminal plasma composition have been shown (20–23).

This work's main objective was to investigate a possible correlation between non-conventional sperm quality parameters, including apoptosis-related markers, capacitation status and oxygen consumption, and field fertility results after artificial insemination (AI) with refrigerated ram semen samples. Moreover, differences in the parameters between samples obtained in the breeding and non-breeding seasons were examined.

## Materials and Methods

### Sperm Collection and Seminal Dose Preparation

All the experiments were performed using semen samples obtained during the course of 3 years from 45 mature *Rasa Aragonesa* rams maintained at the Centro de Transferencia Agroalimentaria (CTA) in Zaragoza, Spain. Samples were obtained throughout the year, but for comparing results we divided the data between breeding (B, September to March) and non-breeding (NB, April to August) seasons. All animal procedures were performed in accordance with the Spanish Animal Protection Regulation RD1201/05, which conforms to European Union Regulation 2010/63. Approval from the Ethics Committee of the University of Zaragoza was not a prerequisite for this study since we worked with the obtained semen samples. The semen samples were collected using an artificial vagina, within the regular collecting schedule at the AI station. A total of 357 different ejaculates were used in this study. Sperm concentration was calculated in duplicate using a Neubauer chamber (Marienfeld, Germany). Progressive individual motility evaluations were assessed subjectively by visual estimation under a phase-contrast microscope at ×100 magnification, maintained at 37°C. All the samples used ranged between 80 and 85% motility. Semen was diluted to 4 × 10^8^ cells/mL and seminal doses of 0.25 mL were prepared in straws and kept at 15°C until insemination time. Two straws of each sample were sent at the same temperature to our laboratory for analysis.

The artificial inseminations were carried out by the technical teams of ANGRA (National Association of *Rasa Aragonesa* Breeding) and UPRA-Grupo Pastores on different farms owned by these cooperative companies. A total of 3,906 ewes from 52 farms were inseminated. Data reported by the cooperatives were fertility (proportion of ewes that lambed per ewes inseminated), fecundity (number of lambs born per ewes inseminated) and prolificacy (number of lambs born per ewe lambed).

From now on these above-mentioned cooperatives will be named as Coop 1 and Coop 2. An essential difference in the reproductive management between both cooperatives was the use of melatonin implants in rams belonging to Coop 2 during the non-breeding season to avoid the reproductive effects of seasonality.

### Preparation of Sperm Samples for Laboratory Analyses

In order to remove the dilution medium, 200 μL of each straw was diluted up to 1 mL with PBS and washed with 7 mL of sucrose buffer (10 mM NaCl, 222 mM sucrose, 2.5 mM KCl, 20 mM HEPES, 10 mM glucose, 1 mg/mL polyvinyl alcohol and polyvinyl pyrrolidone, pH 7.5) by centrifugation at 500 × g for 10 min. The supernatant was removed, leaving 1 mL to resuspend the pellet, and the sperm concentration was calculated using the Neubauer chamber (Marienfeld, Germany).

### Flow Cytometry Analysis

All the measurements were performed on a Beckman Coulter FC 500 (Beckman Coulter Inc., Brea, CA) with CXP software, equipped with two lasers of excitation (Argon ion laser 488 nm and solid-state laser 633 nm) and 5 filters of absorbance (FL1-525, FL2- 575, FL3-610, FL4-675 and FL5-755, ± 5 nm each bandpass filter). A minimum of 20,000 events was recorded in all the experiments. The sperm population was gated for further analysis on the basis of its specific forward (FS) and side scatter (SS) properties; other non-sperm events were excluded. A flow rate stabilized at 200–300 cells/s was used.

#### Evaluation of Sperm Membrane Integrity

To determine plasma membrane integrity, 3 μL of each stain, 1 mM carboxyfluorescein diacetate (CFDA) and 1.5 mM propidium iodide (PI) (both from Sigma-Aldrich, subsidiary of Merck KGaA, Darmstadt, Germany), and 3 μL formaldehyde (0.005% final concentration) were added to 300 μL of sperm samples (final concentration of 5 × 10^6^ cells/mL), based on a modification of the procedure described by Harrison and Vickers (24). Samples were incubated at 37°C in darkness for 15 min. The monitored parameters were FS log, SS log, FL1 (CFDA) and FL4 (PI). For the gated sperm cells, percentages of intact membrane (IM; CFDA+/PI-) spermatozoa were evaluated.

#### Evaluation of the Plasmalemma Stability and Mitochondrial Membrane Potential

A double stain technique (25, 26) was performed with slight modifications, using YO-PRO-1 (1 mM in DMSO) and MitoTracker Deep Red (MitoT 10 μM in DMSO), both from Thermo Fisher Scientific (Waltham, MA, USA). YO-PRO-1 is a DNA dye used as an apoptotic marker because it only permeates into cells that are beginning to undergo apoptosis. MitoTracker is a mitochondrion-selective stain that passively diffuses across the plasma membrane and accumulates in active mitochondria and is used to evaluate mitochondrial membrane potential (ΔΨm). Two μL of each dye were added to 300 μl sperm samples (4 × 10^7^ spermatozoa) that were incubated at RT in darkness for 15 min. The monitored parameters were FS log, SS log, FL1 (YO-PRO-1) and FL5 (MitoT).

The MitoTracker data were analyzed as suggested by Hallap et al. (27) considering spermatozoa with high fluorescence as cells with high ΔΨm. Four different sperm populations were classified according to staining (28): YO-PRO-1 -/MitoT+, non-permeant (NP) membrane cells with high ΔΨm; YO-PRO-1 -/MitoT-, NP cells with low ΔΨm; YO-PRO-1 +/MitoT-, apoptotic cells that have lost the integrity of their plasmalemma and with low ΔΨm; and YO-PRO-1 +/MitoT+, cells that have lost the integrity of their plasmalemma although with high ΔΨm.

#### Detection of Membrane Phosphatidylserine (PS) Translocation

Annexin V is a calcium-dependent phospholipid-binding protein with high affinity for phosphatidylserine (PS). We used the simultaneous staining with FITC- Annexin V (Thermo Fisher Scientific, Waltham, MA, USA) to detect PS translocation and propidium iodide (PI, Sigma-Aldrich, subsidiary of Merck KGaA, Darmstadt, Germany) to differentiate between live and dead cells, with or without PS translocation. Aliquots of 300 μL (4 × 10^6^ cells) were stained with FITC-Annexin V (1 μL) combined with 7.5 μM PI and incubated at 37°C in the dark for 15 min in binding buffer. The monitored parameters were FS log, SS log, FL1 (FITC-Annexin V) and FL4 (PI). Four sperm subpopulations were distinguished: intact membrane (IM) cells with (Annx+/PI-) or without PS translocation (Annx-/PI-); and membrane-damaged cells with (Annx+/PI+) or without PS translocation (Annx-/PI+).

#### Detection of Activated Caspases

The caspase FITC-VAD-FMK *in situ* marker (Vybrant® FAM Caspase-3 and−7 Assay Kit, Thermo Fisher Scientific, Waltham, MA, USA) was used to detect active caspase-3 and−7. This cell-permeable caspase inhibitor peptide conjugated to FITC covalently binds to activated caspases, and functions as an *in situ* marker for apoptosis (29). Samples of 300 μL (1 × 10^6^cells) containing FITC-VADFMK (5 nM) were incubated at 37°C and 5% CO_2_ in the dark for 60 min. After washing twice with 100 μL of washing buffer (supplied with the kit) by centrifuging at 600 × g 8 min at RT, the pellet obtained was resuspended with 300 μL of washing buffer containing 7.3 μM ethidium homodimer (Eth), and flow cytometry was performed within 10 min. The monitored parameters were FS log, SS log, FL1 (FITC-VAD-FMK) and FL4 (Eth). With this technique, four sperm subpopulations were distinguished: intact membrane cells (IM) with (FITC-VAD+/Eth-) or without active caspase-3 and -7 (FITC-VAD-/Eth-); and dead with (FITC-VAD+/Eth+) or without (FITC-VAD-/Eth+) active caspases.

#### Evaluation of DNA Fragmentation

The presence of DNA strand breaks related to apoptosis in spermatozoa was evaluated by the TUNEL (terminal transferase-mediated dUDP nick end-labeling) assay using an *In Situ* Cell Death Detection Kit with fluorescein isothiocyanate (FITC)-labeled dUTP (Sigma-Aldrich, subsidiary of Merck KGaA, Darmstadt, Germany) (30). Sperm samples (4 × 10^7^ cells/mL) were fixed with 4% paraformaldehyde in PBS at RT for 1 h. After two washes with 100 μL of PBS, the samples were permeabilized with 0.1% Triton X-100 in 0.1% sodium citrate. The reaction was performed by incubating the pellet obtained with 50 μL of labeling solution that contained the TdT enzyme and dUTP, for 1 h at 37°C in the dark. For each experimental set, a negative control was prepared by omitting TdT from the reaction mixture. Positive controls were simultaneously prepared by additional treatment with 10 IU DNase I for 10 min at 15–25°C before the labeling reaction. Two subsequent washes with PBS at 600 × g for 10 min at RT were performed to stop the reaction, and flow cytometry analysis was carried out. The monitored parameters were FS log, SS log and FL1 (TUNEL). TUNEL negative spermatozoa were considered DNA-intact.

### Evaluation of the Capacitation Status

The sperm capacitation state was evaluated using the chlortetracycline (CTC) assay (31) that we previously validated for the evaluation of capacitation and acrosome reaction- like changes in ram spermatozoa (32). For staining, washed samples were diluted with PBS (4 × 10^7^ cells/mL). CTC solution (750 mM, Sigma-Aldrich) was prepared daily in a buffer containing 20 mM Tris, 130 mM NaCl and 5 mM cysteine, pH 7.8, and passed through a 0.22 mm filter (Merck KGaA, Darmstadt, Germany). To 18 mL of sperm sample, 2 mL of ethidium homodimer-1 (EthD-1, 23.3 mM) were added and then incubated at 37°C in the dark for 10 min. Thereafter, 20 mL of CTC solution and 5 mL of 12.2% (w/v) paraformaldehyde in 0.5 M Tris–HCl, pH 7.8, were added. An aliquot of 4 mL of stained sample was placed onto a glass slide at room temperature, and mixed with 2 mL of 0.22 M Triethylenediamine (an antifade-reagent, Merck KGaA, Darmstadt, Germany) in glycerol:phosphate buffered saline (PBS, 9:1). The samples were covered with 24 × 48 mm coverslips, sealed with colorless enamel, and stored at 4°C in the dark.

For the evaluation of CTC patterns, the samples were observed under a Nikon Eclipse E-400 microscope with epifluorescence illumination using a V-2A filter at 1000 X magnification. At least 200 cells were counted in duplicate for each sample. Spermatozoa were classified into one of the following patterns (33): non-capacitated (NC, even distribution of fluorescence on the head, with or without a bright equatorial band), capacitated (C, with fluorescence in the anterior portion of the head) and acrosome-reacted cells (showing no fluorescence on the head). The use of ethidium homodimer in this staining allows differentiating live and dead spermatozoa in these three types.

### Determination of Oxygen Consumption

Sperm oxygen consumption was measured with a Clark oxygen electrode linked to a recorder system software (Oxygraph, Hansatech Instruments Ltd., Norfolk UK), as previously described (13). The zero point was set by adding sodium dithionite to the chamber filled with distilled water at 37°C and maintained with constant stirring to ensure a homogeneous distribution of O_2_. Sperm samples were diluted with PBS up to 3 × 10^7^ cells/mL and 1 mL of the sperm solution was loaded into the pre-warmed chamber. The plunger was inserted to expel air, and O_2_ consumption was monitored for 3 min at 37°C with constant stirring. Results were expressed as fmol O_2_·mL^−1^·min^−1^ per million cells.

### Statistical Analysis

Statistical analyses were undertaken using the IBM SPSS Statistics software v. 21 (Armonk, NY, USA) and GraphPad Prism v.9.0.0 for Windows (GraphPad Software, San Diego, CA, USA).

Normality of the data was analyzed with the Kolmogorov-Smirnov test. Correlations between tested sperm parameters and field fertility were calculated using the Spearman rank correlation method. The Mann-Whitney test was carried out to determine whether there were significant differences between groups in some of the above-mentioned parameters. Finally, multiple logistic regression analysis was used for determining the dichotomous field fertility rate (higher or lower than the mean fertility) with sperm parameters. Due to the unbalanced data registry, only the parameters that allowed us to have more than 100 observations in the logistic regression analysis were included in the model.

## Results

### Sperm Quality and Reproductive Parameters of Refrigerated Samples

The mean values of seminal quality and the reproductive parameters obtained with refrigerated seminal doses are summarized in Table 1. When these refrigerated samples reached our laboratory, and after sucrose buffer washing, the mean percentage of intact membrane (IM) spermatozoa, estimated as PI- was around 50% (47.63 ± 1.20%). However, percentages of IM spermatozoa that remained in a non-capacitated state (IM-NC), or without phosphatidylserine translocation or caspases activation, were relatively lower (19.12 ± 0.61%, 36.71 ± 0.90%, and 32.82 ± 1.05%, respectively). When the mitochondrial membrane potential was evaluated together with membrane permability, the percentage of non-permeant cells with high inner membrane potential (ΔΨm) was also low (27.33 ± 1.39%). Regarding other apoptotic parameters, the mean value of spermatozoa without DNA injury in all evaluated seminal doses was 84.87 ± 0.44% (Table 1A). The oxygen consumption by these refrigerated samples was 0.59 ± 0.12 fmol O_2_·mL^−1^ min^−1^/10^6^ cells.

**Table 1:** Mean, minimum and maximum values of (A) all analyzed parameters in refrigerated seminal doses, and (B) reproductive results obtained after artificial insemination with these seminal doses.

	***n***	**Mean**	**S.E.M**	**Minimum**	**Maximum**
**A) Sperm quality parameters**					
Intact membrane (IM) spermatozoa (PI-) (%)	286	47.63	1.20	3.00	95.00
**IM non-capacitated (IM-NC) spermatozoa (%)**	357	19.12	0.61	0.00	55.00
Non permeant (NP) sperm with high ΔΨm (YO-PRO-1-/ MitoT+) (%)	138	27.33	1.39	0.10	68.90
IM sperm without PS translocation (Annx-/PI-) (%)	346	36.71	0.90	0.2	85.20
IM sperm without active caspases (FITC-VAD-/Eth-) (%)	277	32.82	1.05	0.00	78.50
DNA-intact spermatozoa (TUNEL-) (%)	357	84.87	0.44	50.00	99.00
Oxygen consumption (fmol O_2_·mL^−1^·min^−1^ per million cells)	203	0.59	0.12	0.09	1.04
**B) Reproductive parameters**					
Fertility (ewes lambing per ewes inseminated, %)	357	51.97	1.10	0.00	100.00
Fecundity (number of lambs born per ewes inseminated)	357	0.83	0.20	0.00	2.14
Prolificacy (number of lambs born per ewe lambing)	357	1.56	0.20	0.00	3.00

*N indicates the number of seminal doses evaluated and S.E.M. is the standard error of the mean*.

The reproductive results obtained after cervical inseminations were 51.97 ± 1.1% fertility, 0.83 ± 0.2 fecundity and 1.56 ± 0.2 prolificacy (Table 1B).

### Influence of Season on the Analyzed Parameters

Comparing the results obtained in the breeding (B) and non-breeding (NB) season, we found significant differences in some of the analyzed sperm quality parameters (Table 2). The mean value of IN (PI-) spermatozoa was almost 10% higher in the B than in the NB season, this difference being highly significant (*P* = 0.001, Table 2). Regarding apoptotic-like markers, the percentage of NP spermatozoa with high ΔΨm and IM sperm without PS translocation was also higher in the B than in the NB season (*P* < 0.001 and *P* = 0.05, respectively).

**Table 2:** Differences in analyzed parameters between seminal doses from the breeding and the non-breeding season.

**Parameter**	**Breeding season**	**Non-breeding season**	**Significance level (*P*)**
Intact membrane (IM) spermatozoa (PI-) (%)	51.97 ± 1.64	42.57 ± 1.66	*P =* 0.001
IM non-capacitated (IM-NC) spermatozoa (%)	19.92 ± 0.82	18.07 ± 0.90	*P =* 0.087
Non-permeant (NP) sperm with high ΔΨm (YO-PRO-1 -/MitoT+) (%)	36.68 ± 1.77	16.21 ± 1.12	*P* < 0.001
IM sperm without PS translocation (Annx-/PI-) (%)	39.06 ± 1.16	33.82 ± 1.39	*P =* 0.05
IM sperm without active caspases (FITC-VAD-/Eth-) (%)	32.07 ± 1.18	33.63 ± 1.79	n.s.
DNA-intact spermatozoa (TUNEL-) (%)	85.17 ± 0.59	84.49 ± 0.66	n.s.
Oxygen consumption (fmol O_2_·mL^−1^·min^−1^ per million cells)	0.55 ± 0.17	0.61 ± 0.17	*P =* 0.019
Fertility (ewes lambing per ewes inseminated, %)	53.01± 1.42	50.63 ± 1.72	n.s.
Fecundity (number of lambs born per ewes inseminated)	0.86 ± 0.02	0.79 ± 0.03	n.s.
Prolificacy (number of lambs born per ewe lambing)	1.59 ± 0.02	1.53 ± 0.03	n.s.

*Results are expressed as a mean value ± standard error of the mean. Last column reflects the level of significance. n.s., non significant*.

Moreover, spermatozoa in samples from the NB season showed significantly higher oxygen consumption than those in samples from the B season (*P* < 0.05). Regarding capacitation, the percentage of IM-NC spermatozoa was slightly higher in B than in NB, with a tendency to significance (*P* = 0.087).

Differences in the above-mentioned parameters did not lead to significant differences in the reproductive results between the two seasons. However, fertility, fecundity and prolificacy data were slightly higher in B than in NB (Table 2).

### Differences Between Sires and Reproductive Management

The rams used in this study belonged to two farming cooperatives, named here as Coop 1 and Coop 2, so we also compared sperm quality parameters between seminal doses from animals belonging to both companies (Table 3). Percentages of IM (PI-) and IM without PS translocation spermatozoa were significantly higher in samples from Coop 1 than Coop 2 (*P* < 0.001 and *P* < 0.05, respectively). In contrast, the Coop 2 seminal doses showed a higher value of DNA-intact spermatozoa (*P* < 0.001) than those from Coop 1. However, these differences did not lead to significant dissimilarities in the reproductive results of the two cooperatives (Table 3).

**Table 3:** Differences in analyzed parameters between cooperatives.

**Parameter**	**COOP 1**	**COOP 2**	**Significance level (P)**
Intact membrane (IM) spermatozoa (PI-) (%)	50.68 ± 1.50	41.41 ± 1.84	*P* < 0.001
IM non-capacitated (IM-NC) spermatozoa (%)	19.36 ± 0.74	18.45 ± 1.01	n.s.
Non-permeant (NP) sperm with high ΔΨm (YO-PRO-1-/MitoT+) (%)	26.08 ± 1.57	31.65± 2.95	n.s
IM sperm without PS translocation (Annx-/PI-) (%)	37.92 ± 1.06	33.47 ± 1.70	*P =* 0.024
IM sperm without active caspases (FITC-VAD-/Eth-) (%)	33.81 ± 1.29	30.97 ± 1.79	n.s.
DNA-intact spermatozoa (TUNEL-) (%)	83.63 ± 0.53	88.21 ± 0.66	*P* < 0.001
Oxygen consumption (fmol O_2_·mL^−1^·min^−1^ per million cells)	0.59 ± 0.14	0.58 ± 0.22	n.s.
Fertility (ewes lambing per ewes inseminated, %)	51.37 ± 1.28	53.60 ± 2.12	n.s.
Fecundity (number of lambs born per ewes inseminated)	0.82 ± 0.02	0.84 ± 0.03	n.s.
Prolificacy (number of lambs born per ewe lambing)	1.56 ± 0.02	1.56 ± 0.03	n.s.

*Results are expressed as a mean value ± standard error of the mean. Last column reflects the level of significance. n.s., non significant*.

An important difference in the reproductive management between the cooperatives was the use of melatonin implants in rams belonging to Coop 2 during the NB season, in order to avoid the reproductive effects of seasonality. To investigate whether the observed differences between the two cooperatives were due to the melatonin treatment during the NB season, we compared the sperm quality values between cooperatives in the B and NB seasons. In NB, only the percentages of spermatozoa with intact DNA were significantly higher in Coop 2 (implanted animals) than in Coop 1 (non-implanted) (88.72 ± 0.96 vs. 83.11 ± 0.79%, *P* < 0.001, respectively). However, differences in this parameter were also significant (*P* < 0.001) in the B season (Table 4).

**Table 4:** Differences in analyzed parameters between seminal doses from the breeding and the non-breeding season for both cooperatives.

	**Breeding season**		**Non-Breeding season**	
**Parameter**	**COOP 1**	**COOP 2**	**COOP 1**	**COOP 2**
Intact membrane (IM) spermatozoa (PI-) (%)	56.71 ± 2.05	43.68 ± 2.40^***^	44.40 ± 2.02	38.06 ± 2.82
IM non-capacitated (IM-NC) spermatozoa (%)	20.23 ± 1.08	19.15 ± 1.01	18.23 ± 1.00	17.36 ± 2.07
Non-permeant (NP) sperm with high ΔΨm (YO-PRO-1 -/MitoT+) (%)	37.90 ± 2.34	34.61 ± 2.69	16.82 ± 1.11	n.d.
IM sperm without PS translocation (Annx-/PI-) (%)	40.72 ± 1.34	35.05 ± 2.19^*^	34.68 ± 1.62	31.16 ± 2.68
IM sperm without active caspases (FITC-VAD-/Eth-) (%)	33.23 ± 1.55	30.39 ± 1.81	32.45 ± 1.19	33.79 ± 2.05
DNA-intact spermatozoa (TUNEL-) (%)	84.06 ± 0.73	87.87 ± 0.90^***^	83.11 ± 0.79	88.72 ± 0.96^***^
Oxygen consumption (fmol O_2_·mL^−1^·min^−1^ per million cells)	0.56 ± 0.02	0.55 ± 0.02	0.61 ± 0.18	0.62 ± 0.42
Fertility (ewes lambing per ewes inseminated, %)	52.19 ± 1.72	54.99 ± 2.53	50.15 ± 1.96	51.46 ± 3.76
Fecundity (number of lambs born per ewes inseminated)	0.85 ± 0.03	0.88 ± 0.05	0.79 ± 0.03	0.79 ± 0.06
Prolificacy (number of lambs born per ewe lambing)	1.60 ± 0.03	1.57 ± 0.04	1.53 ± 0.03	1.55 ± 0.06

*Results are expressed as a mean value ± standard error of the mean*.

* *P < 0.05 and*

*** *P < 0.001 between cooperatives within season. n.d, not enough data to include in the comparative analysis*.

### Relationship Between Reproductive and Sperm Quality Parameters

When the relationship between reproductive and sperm quality parameters was evaluated, only the percentage of IM-NC (intact membrane, non-capacitated) spermatozoa showed a significant correlation with field fertility (*P* = 0.005) and fecundity (*P* = 0.007), independently of the season, the cooperative and the distance (Table 5). When the effect of the season was evaluated, the percentage of IM-NC spermatozoa showed significant correlation with all the *in vivo* reproductive results during the NB season (*P* = 0.025, *P* = 0.004, and *P* = 0.007 for fertility, fecundity and prolificacy, respectively), but only a tendency to significance with fertility (*P* = 0.082) during the B season.

**Table 5:** Spearman's rank correlation coefficient (Spearman's ρ) between some analyzed sperm parameters and the reproductive results obtained after artificial insemination, considering overall data and data separated by season or farm distance to the semen collection point.

	**Parameter**	**Fertility**	**Fecundity**	**Prolificacy**
Overall data	IM non-capacitated (IM-NC) spermatozoa (%)	0.147^**^	0.143^**^	n.s
In B season	IM non-capacitated (IM-NC) spermatozoa (%)	0.122^†^	n.s.	n.s.
	Oxygen consumption (fmol O_2_·mL^−1^·min^−1^ per million cells)	0.245^*^	0.346^**^	0.212^*^
	NP sperm with high ΔΨm (YO-PRO-1-/MitoT+) (%)	0.246^*^	0.291^*^	n.s.
	IM sperm without active caspases (FITC-VAD-/Eth-) (%)	n.s.	0.163^*^	n.s.
In NB season	IM non-capacitated (IM-NC) spermatozoa (%)	0.180^*^	0.232^**^	0.191^*^
Distance <50 km	IM non-capacitated (IM-NC) spermatozoa (%)	n.s.	n.s.	n.s
Distance 50–100 km	IM non-capacitated (IM-NC) spermatozoa (%)	0.195^*^	0.176^†^	n.s.
Distance >100 km	IM non-capacitated (IM-NC) spermatozoa (%)	0.158^*^	0.188^*^	0.146^†^
	DNA-intact spermatozoa (TUNEL-) (%)	0.144^†^	n.s.	n.s.

*n.s, non significant;*

† *P < 0.1;*

* *P < 0.05; and*

** *P < 0.01. B, breeding; NB, non-breeding; IM, Intact membrane; NP, non permeant membrane*.

All seminal doses were prepared in the same AI centre and used in different farms distributed in a 250 km radius. The data were then divided in three categories according to the distance from the collection centre to the farm where the artificial insemination was carried out: short (<50 km), medium (50–100 km) and long (>100 km) distance. When the data were sorted by distance, the correlation between the percentage of IM-NC spermatozoa and reproductive parameters increased progressively with remoteness (Table 5).

Furthermore, three other sperm quality characteristics showed significant correlation with the reproductive parameters, but only in the B season: the oxygen consumption correlated significantly with fertility (*P* < 0.05), fecundity (*P* < 0.01) and prolificacy (*P* < 0.05); the percentage of spermatozoa with NP membrane and high ΔΨm (YO-PRO-1-/MitoT+) with fertility and fecundity (*P* < 0.05); and the percentage of IM spermatozoa without active caspases with fecundity (*P* < 0.05). When the data were divided into categories according to the farm distance, TUNEL negative cells showed a positive correlation with fertility (*P* < 0.1) when the distance was >100 km.

**Figure 1:**
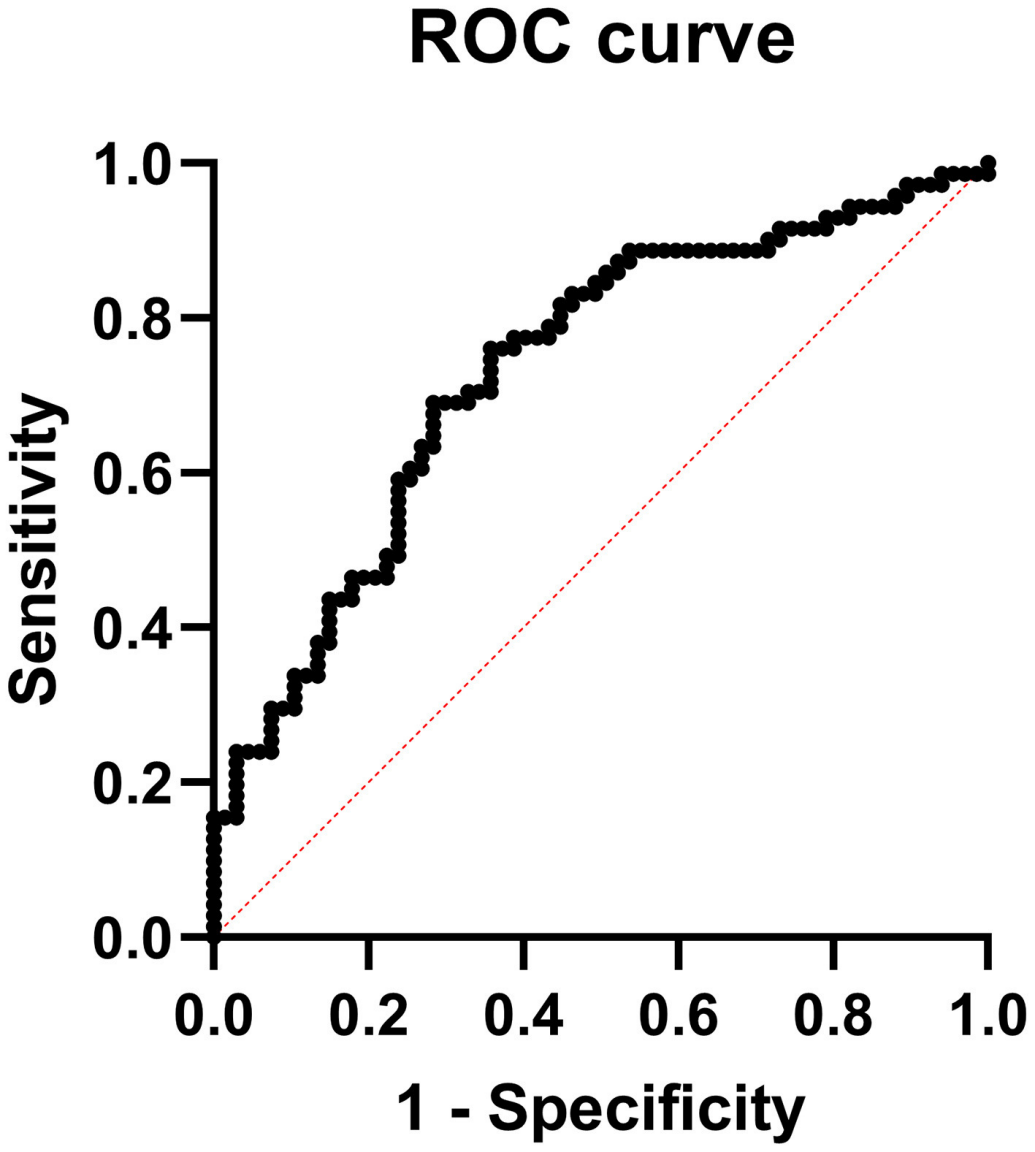
Receiver operating characteristic (ROC) curve analysis of prediction model for fertility >52% after AI with refrigerated ram seminal doses.

Multiple logistic regression analysis revealed that the increment in percentage of IM-NC (*P* = 0.02) together with the rise in DNA-intact spermatozoa (*P* = 0.04), would increase the probability of obtaining a fertility higher than the mean (>52%) (Table 6). ROC analysis (Figure 1) was used to identify the accuracy (area under the curve, which indicates discriminatory ability), sensitivity (true positive rate) and specificity (true negative rate) of the model. In our model, the area under the curve was 0.7328 (*P* < 0.001) and sensitivity and specificity were 70.42 and 64.18%, respectively. The positive and negative predictive power was 67.57 and 67.19%, respectively.

**Table 6:** Multiple logistic regression results of fertility >52% (*n* = 137).

**Variable**	**Beta**	**Odds ratios**	**95% CI for OR**		***P*-value**
			**Lower limit**	**Upper limit**	
Intercept	−9.478	7.65 × 10^−5^	1.38 × 10^−7^	0.02	0.002
Distance [<50 km]	–	1.000	–	–	–
Distance [50–100 km]	−0.917	2.503	0.865	7.516	0.09
Distance [>100 km]	1.207	3.344	1.314	9.018	0.01
Cooperative [Coop 1]	–	1.000	–	–	–
Cooperative [Coop 2]	0.044	1.045	0.349	3.189	0.94
Season [breeding]	–	1.000	–	–	–
Season [non-breeding]	0.844	2.325	0.778	7.345	0.14
Intact membrane (IM) spermatozoa (PI-) (%)	0.041	1.042	0.998	1.091	0.07
IM sperm without PS translocation (Annx-/PI-) (%)	−0.027	0.973	0.928	1.018	0.24
IM non-capacitated (IM-NC) spermatozoa (%)	0.056	1.058	1.011	1.110	0.02
DNA-intact spermatozoa (TUNEL-) (%)	0.061	1.063	1.006	1.129	0.04
IM sperm without active caspases (FITC-VAD-/Eth-) (%)	0.009	1.009	0.982	1.037	0.51
Non-permeant (NP) sperm with high ΔΨm (YO-PRO-1-/ MitoT+) (%)	0.023	1.024	0.994	1.056	0.12

*OR, Odds ratio; CI, confidence interval; (-) not applicable as the reference (most frequent value)*.

## Discussion

In this study, we determined the relationship between *in vivo* fertility and some sperm quality parameters, including membrane integrity, capacitation state, mitochondria functionality, metabolic activity and apoptotic-like markers, in rams. We found that, when all data was evaluated, only one marker significantly correlated with *in vivo* fertility and with fecundity. This parameter was the percentage of intact membrane non-capacitated (IM-NC) spermatozoa, evaluated using the chlortetracycline (CTC) assay in combination with ethidium homodimer. We had previously validated this procedure for the evaluation of capacitation and acrosome reaction-like changes in ram spermatozoa (32) following a procedure reported earlier (34). This assay allows us to estimate simultaneously changes in the intracellular calcium distribution related to the capacitation status and plasma membrane integrity. Although some authors have also reported a correlation between capacitation status and *in vivo* fertility, their studies were conducted with frozen-thawed semen in other species (35, 36). However, to the best of our knowledge, this is the first study that correlates capacitation status with field fertility using refrigerated ram semen. Capacitation is a prerequisite for fertilization, but it has to occur in the female reproductive tract near the oocyte. If a high number of spermatozoa undergo capacitation during cryopreservation and transport, they will be less likely to survive until the time of insemination.

When the data from the seminal samples were divided into three categories according to the distance from the AI centre where the seminal doses were prepared to each farm, the relationship between the percentage of IM-NC spermatozoa and the reproductive results was lost for the nearest farms and increased progressively with remoteness. Furthermore, the percentage of spermatozoa without DNA damage also showed a tendency to significance in its positive correlation with field fertility when inseminations were done on remote farms. These results suggest that these parameters, the percentage of IM-NC and DNA-intact spermatozoa could be useful indicators of the sperm resistance over time. The increment of both the percentage of IM-NC and the DNA-intact spermatozoa would increase the probability of obtaining a fertility higher than the mean, as revealed by a multiple logistic regression analysis.

Sheep are a seasonal, short-day breeder species. Thus, when we compared sperm quality parameters between the breeding (B) and non-breeding (NB) seasons, we found that seminal doses from the B season presented higher percentages of IM spermatozoa, NP with high ΔΨm spermatozoa, and IM without PS translocation sperm than in the NB season. Therefore, we can conclude that there are fewer spermatozoa showing apoptotic-like features in the seminal doses in the B than in the NB season. Although differences in sperm motility, morphology or viability between the two seasons have already been reported (20, 21, 37), this is the first time, to the best of our knowledge, that the effect of the season on apoptotic-like markers in ram ejaculates has been revealed. However, although fertility, fecundity and prolificacy results were slightly higher in the B than in the NB season, these differences in apoptotic-like parameters did not lead to significant differences in the reproductive results between the two seasons. Nonetheless, the correlation between the percentage of IM-NC spermatozoa and all the reproductive parameters, including prolificacy, increased when this was evaluated separately in the NB season data. This suggests that this marker would be more effective as a predictor of fertility when the sperm samples are “more stressed” or of less quality, as occurred when the time until insemination increased.

Plasma membrane integrity, evaluated as the non-permeability to certain dyes, is usually included in routine sperm analysis. However, this kind of evaluation does not detect subtle modifications in the sperm plasmalemma that reflect essential changes, such as the beginning of apoptosis, which lead the spermatozoa to certain death. The lack of correlation between membrane integrity and *in vivo* fertility has already been reported (2, 38–40) and is consistent with the results of the present study. Other authors found a significant, albeit limited, predictive capacity of this parameter on field fertility (41). However, it is worth pointing out that when PI was combined with an *in situ* activated caspase determination, or when we used more restrictive assays such as the double staining with YO-PRO-1 and Mitotracker Deep Red, which evaluates the beginning of plasmalemma destabilization and the loss of mitochondrial membrane potential, we found a significant correlation with the reproductive results in the B season.

The determination of sperm oxygen consumption using a Clark electrode has been used as a relatively simple method to evaluate the sperm mitochondrial function in several species such as stallion (42), human (43), boar (44), and ram (13). Oxygen consumption has often been considered a good indicator of the overall metabolic activity within cells, including spermatozoa (45, 46), and it has also been correlated with fertility in bulls (17). In this study, a positive correlation between this parameter and reproductive results was found, but only in the B season. Surprisingly, in samples from the NB season, the oxygen consumption was significantly higher than in those from the B season. This could be explained by the existence of a higher number of cells with mitochondrial membrane damage that could cause the membranes to become more “leaky” to protons, thereby increasing the oxygen consumption required for the maintenance of the proton gradient across the mitochondrial membrane and ATP synthesis (17).

We must highlight that the sires used in this study belonged to two farming cooperatives with different breeding schemes. Seminal doses from Coop 1, although presenting significantly higher membrane integrity values, also had higher DNA damage values than those from Coop 2. These results could be influenced by the use of melatonin implants in the NB season by Coop 2. We have already proved that melatonin implants during the NB season modify ram sperm motility characteristics and seem to improve fertilization results (47). However, in this study we found differences in sperm quality between the cooperatives in both seasons, not only during NB. Therefore, these differences could not be accurately attributed to the melatonin treatment. Furthermore, they did not lead to significant variations in the reproductive results achieved for both cooperatives.

In conclusion, this study reveals, for the first time, differences in apoptotic-like markers and oxygen consumption in ram seminal AI doses between the B and NB seasons. Moreover, we found two markers, the percentage of membrane intact non-capacitated spermatozoa, and the percentage of DNA-intact spermatozoa, that would allow discriminating between high and low fertility semen samples. Thus, these markers could be used to discard ejaculates with low fertilizing capacity and to test the value of particular males as semen donors after analyzing an appropriate number of their ejaculates. Both procedures might improve fertility results obtained by artificial insemination in ovine.

## Data Availability Statement

The datasets generated for this study can be found in the figshare repository (doi: 10.6084/m9.figshare.13521647).

## Ethics Statement

All animal procedures were performed in accordance with the Spanish Animal Protection Regulation RD1201/05, which conforms to European Union Regulation 2010/63. Approval from the Ethics Committee of the University of Zaragoza was not a prerequisite for this study since we worked with the obtained semen samples. Written informed consent for participation was not obtained from the owners because ewes from farms associate to cooperatives were inseminated as always according to the breeding schedule by each cooperative, without any experimental procedure. Seminal doses for artificial insemination were collected at the Centro de Transferencia Agroalimentaria (CTA) in Zaragoza from rams belonging to cooperatives. We only analyzed in the lab an aliquot of the same seminal doses used for artificial inseminations, and veterinarians working for the cooperatives provide us results of fertility in order to study possible correlations.

## Author Contributions

RP-P and TM-B: conceptualization, experimental approach, and writing—original draft preparation. NM and JD: sperm quality analysis. FQ: semen samples preparation. AL and EF: responsible for artificial inseminations in farms. AC and NM: statistical analysis. AC and RP-P: data curation, writing—review, and editing. RP-P, JC-P, and TM-B: supervision. TM-B: project administration. TM-B and JC-P: funding acquisition. All authors contributed to the article and approved the submitted version.

## Conflict of Interest

The authors declare that the research was conducted in the absence of any commercial or financial relationships that could be construed as a potential conflict of interest.

## References

[1] Jeyendran RS Caroppo E Rouen A Anderson A Puscheck E. Selecting the most competent sperm for assisted reproductive technologies. Fertil Steril. (2019) 111:851–63. 10.1016/j.fertnstert.2019.03.024 31029239

[2] Meara CMO Hanrahan JP Prathalingam NS Owen JS Donovan A Fair S. Relationship between *in vitro* sperm functional tests and *in vivo* fertility of rams following cervical artificial insemination of ewes with frozen-thawed semen. Theriogenology. (2008) 69:513–22. 10.1016/j.theriogenology.2007.12.003 18248736

[3] Rodríguez-Martínez H. Can we increase the estimated value of semen assessment? Reprod Domest Anim. (2006) 41(Suppl. 2):2–10. 10.1111/j.1439-0531.2006.00764.x 16984464

[4] Baro Graf C Ritagliati C Torres-Monserrat V Stival C Carizza C Buffone MG. Membrane potential assessment by fluorimetry as a predictor tool of human sperm fertilizing capacity. Front Cell Dev Biol. (2020) 7:383. 10.3389/fcell.2019.00383 32010695 PMC6979052

[5] Dutta S Majzoub A Agarwal A. Oxidative stress and sperm function: A systematic review on evaluation and management. Arab J Urol. (2019) 17:87–97. 10.1080/2090598X.2019.1599624 31285919 PMC6600059

[6] Amann RP Hammerstedt RH. *In-vitro* evaluation of sperm quality - an opinion. J Androl. (1993) 14:397–406. 8294222

[7] Kumaresan A Johannisson A Al-Essawe EM Morrell JM. Sperm viability, reactive oxygen species, and DNA fragmentation index combined can discriminate between above- and below-average fertility bulls. J Dairy Sci. (2017) 100:5824–36. 10.3168/jds.2016-12484 28478003

[8] Sellem E Broekhuijse ML Chevrier L Camugli S Schmitt E Schibler L. Use of combinations of *in vitro* quality assessments to predict fertility of bovine semen. Theriogenology. (2015) 84:1447–54.e1445. 10.1016/j.theriogenology.2015.07.035 26296523

[9] Fuller SJ Whittingham DG. Capacitation-like changes occur in mouse spermatozoa cooled to low temperatures. Mol Reprod Dev. (1997) 46:318–24. 10.1002/(SICI)1098-2795(199703)46:3<318::AID-MRD10>3.0.CO;2-V 9041134

[10] Gangwar C Kharche SD Mishra AK Saraswat S Kumar N Sikarwar AK. Effect of diluent sugars on capacitation status and acrosome reaction of spermatozoa in buck semen at refrigerated temperature. Trop Anim Health Prod. (2020) 52:3409–15. 10.1007/s11250-020-02374-8 32918161

[11] Paulenz H Söderquist L Pérez-Pé R Andersen Berg K. Effect of different extenders and storage temperatures on sperm viability of liquid ram semen. Theriogenology. (2002) 57:823–36. 10.1016/S0093-691X(01)00683-5 11991386

[12] Vadnais ML Roberts KP. Effects of seminal plasma on cooling-induced capacitative changes in boar sperm. J Androl. (2007) 28:416–22. 10.2164/jandrol.106.001826 17192595

[13] Del Valle I Mendoza N Casao A Cebrian-Perez JA Perez-Pe R Muino-Blanco T. Significance of non-conventional parameters in the evaluation of cooling-induced damage to ram spermatozoa diluted in three different media. Reprod Domest Anim. (2010) 45:e260–8. 10.1111/j.1439-0531.2009.01552.x 19930134

[14] Gallardo Bolaños JM Miró Morán Á Balao da Silva CM Morillo Rodríguez A Plaza Dávila M Aparicio IM. Autophagy and apoptosis have a role in the survival or death of stallion spermatozoa during conservation in refrigeration. PLoS ONE. (2012) 7:e30688. 10.1371/journal.pone.0030688 22292020 PMC3266901

[15] Mendoza N Casao A Pérez-Pé R Cebrián-Pérez JA Muiño-Blanco T. New insights into the mechanisms of ram sperm protection by seminal plasma proteins. Biol Reprod. (2013) 88:1–15. 10.1095/biolreprod.112.105650 23636812

[16] Zhang J Nuebel E Wisidagama DR Setoguchi K Hong JS Van Horn CM. Measuring energy metabolism in cultured cells, including human pluripotent stem cells and differentiated cells. Nat Protoc. (2012) 7:1068–85. 10.1038/nprot.2012.048 22576106 PMC3819135

[17] Garrett LJA Revell SG Leese HJ. Adenosine triphosphate production by bovine spermatozoa and its relationship to semen fertilizing ability. J Androl. (2008) 29:449–58. 10.2164/jandrol.107.003533 18046050

[18] Aller JF Aguilar D Vera T Almeida GP Alberio RH. Seasonal variation in sexual behavior, plasma testosterone and semen characteristics of Argentine Pampinta and Corriedale rams. Span J Agric Res. (2012) 10:345–52. 10.5424/sjar/2012102-389-11

[19] Zamiri MJ Khalili B Jafaroghli M Farshad A. Seasonal variation in seminal parameters, testicular size, and plasma testosterone concentration in Iranian Moghani rams. Small Ruminant Res. (2010) 94:132–6. 10.1016/j.smallrumres.2010.07.013

[20] D'alessandro AG Martemucci G. Evaluation of seasonal variations of semen freezability in Leccese ram. Anim Reprod Sci. (2003) 79:93–102. 10.1016/S0378-4320(03)00113-1 12853182

[21] Gundogan M Serteser M. Some reproductive parameters and biochemical properties in Akkaraman and Awassi rams. Turk J Vet Anim Sci. (2005) 29:595–9.

[22] Marti E Mara L Marti JI Muino-Blanco T Cebrian-Perez JA. Seasonal variations in antioxidant enzyme activity in ram seminal plasma. Theriogenology. (2007) 67:1446–54. 10.1016/j.theriogenology.2007.03.002 17433428

[23] Perez-Pe R Barrios B Muino-Blanco T Cebrian-Perez JA. Seasonal differences in ram seminal plasma revealed by partition in an aqueous two-phase system. J Chromatogr B. (2001) 760:113–21. 10.1016/S0378-4347(01)00259-6 11522053

[24] Harrison RA Vickers SE. Use of fluorescent probes to assess membrane integrity in mammalian spermatozoa. J Reprod Fertil. (1990) 88:343–52. 10.1530/jrf.0.0880343 1690300

[25] Del Valle I Gomez-Duran A Holt WV Muino-Blanco T Cebrian-Perez JA. Soy lecithin interferes with mitochondrial function in frozen-thawed ram spermatozoa. J Androl. (2012) 33:717–25. 10.2164/jandrol.111.014944 22134371

[26] Martinez-Pastor F Aisen E Fernandez-Santos MR Esteso MC Maroto-Morales A Garcia-Alvarez O. Reactive oxygen species generators affect quality parameters and apoptosis markers differently in red deer spermatozoa. Reproduction. (2009) 137:225–35. 10.1530/REP-08-0357 19028926

[27] Hallap T Nagy S Jaakma U Johannisson A Rodriguez-Martinez H. Mitochondrial activity of frozen-thawed spermatozoa assessed by mitotracker deep red 633. Theriogenology. (2005) 63:2311–22. 10.1016/j.theriogenology.2004.10.010 15826692

[28] Garcia-Alvarez O Maroto-Morales A Ramon M del Olmo E Montoro V Dominguez-Rebolledo AE. Analysis of selected sperm by density gradient centrifugation might aid in the estimation of *in vivo* fertility of thawed ram spermatozoa. Theriogenology. (2010) 74:979–88. 10.1016/j.theriogenology.2010.04.027 20580077

[29] Wlodkowic D Telford W Skommer J Darzynkiewicz Z. Apoptosis and beyond: cytometry in studies of programmed cell death. Methods Cell Biol. (2011) 103:55–98. 10.1016/B978-0-12-385493-3.00004-8 21722800 PMC3263828

[30] Li X Darzynkiewicz Z. Labelling DNA strand breaks with BrdUTP. Detection of apoptosis and cell proliferation. Cell Proliferation. (1995) 28:571–9. 10.1111/j.1365-2184.1995.tb00045.x 8555370

[31] Ward CR Storey BT. Determination of the time course of capacitation in mouse spermatozoa using a chlortetracycline fluorescence assay. Dev Biol. (1984) 104:287–96. 10.1016/0012-1606(84)90084-8 6745485

[32] Grasa P Cebrián-Pérez JA Muiño-Blanco T. Signal transduction mechanisms involved in *in vitro* ram sperm capacitation. Reproduction. (2006) 132:721–32. 10.1530/rep.1.00770 17071773

[33] Gillan L Evans G Maxwell WM. Capacitation status and fertility of fresh and frozen-thawed ram spermatozoa. Reprod Fertil Dev. (1997) 9:481–487. 10.1071/R96046 9418976

[34] Pérez-Pe R Grasa P Fernandez-Juan M Peleato ML Cebrián-Pérez JA Muiño-Blanco T. Seminal plasma proteins reduce protein tyrosine phosphorylation in the plasma membrane of cold-shocked ram spermatozoa. Mol Reprod Dev. (2002) 61:226–33. 10.1002/mrd.1152 11803559

[35] Kuroda K Fukushima M Harayama H. Premature capacitation of frozen-thawed spermatozoa from subfertile Japanese black cattle. J Reprod Dev. (2007) 53:1079–86. 10.1262/jrd.19031 17615445

[36] Thundathil J Gil J Januskauskas A Larsson B Soderquist L Mapletoft R. Relationship between the proportion of capacitated spermatozoa present in frozen-thawed bull semen and fertility with artificial insemination. Int J Androl. (1999) 22:366–73. 10.1046/j.1365-2605.1999.00194.x 10624605

[37] Marti JI Aparicio IM Leal CL Garcia-Herreros M. Seasonal dynamics of sperm morphometric subpopulations and its association with sperm quality parameters in ram ejaculates. Theriogenology. (2012) 78:528–41. 10.1016/j.theriogenology.2012.02.035 22626774

[38] Gadea J Selles E Marco MA. The predictive value of porcine seminal parameters on fertility outcome under commercial conditions. Reprod Domest Anim. (2004) 39:303–8. 10.1111/j.1439-0531.2004.00513.x 15367261

[39] Perez-Pe R Marti JI Sevilla E Fernandez-Sanchez M Fantova E Altarriba J. Prediction of fertility by centrifugal countercurrent distribution (CCCD) analysis: correlation between viability and heterogeneity of ram semen and field fertility. Reproduction. (2002) 123:869–75. 10.1530/reprod/123.6.869 12052241

[40] Rodriguez-Martinez H. Laboratory semen assessment and prediction of fertility: still utopia? Reprod Domest Anim. (2003) 38:312–8. 10.1046/j.1439-0531.2003.00436.x 12887570

[41] Santolaria P Vicente-Fiel S Palacín I Fantova E Blasco ME Silvestre MA. Predictive capacity of sperm quality parameters and sperm subpopulations on field fertility after artificial insemination in sheep. Anim Reprod Sci. (2015) 163:82–8. 10.1016/j.anireprosci.2015.10.001 26507945

[42] Schober D Aurich C Nohl H Gille L. Influence of cryopreservation on mitochondrial functions in equine spermatozoa. Theriogenology. (2007) 68:745–54. 10.1016/j.theriogenology.2007.06.004 17644168

[43] Ferramosca A Focarelli R Piomboni P Coppola L Zara V. Oxygen uptake by mitochondria in demembranated human spermatozoa: a reliable tool for the evaluation of sperm respiratory efficiency. Int J Androl. (2008) 31:337–45. 10.1111/j.1365-2605.2007.00775.x 17573845

[44] Ramio-Lluch L Fernandez-Novell JM Pena A Colas C Cebrian-Perez JA Muino-Blanco T. “*In vitro*” capacitation and acrosome reaction are concomitant with specific changes in mitochondrial activity in boar sperm: evidence for a nucleated mitochondrial activation and for the existence of a capacitation-sensitive subpopulational structure. Reprod Domest Anim. (2011) 46:664–73. 10.1111/j.1439-0531.2010.01725.x 21121968

[45] Darr CR Cortopassi GA Datta S Varner DD Meyers SA. Mitochondrial oxygen consumption is a unique indicator of stallion spermatozoal health and varies with cryopreservation media. Theriogenology. (2016) 86:1382–92. 10.1016/j.theriogenology.2016.04.082 27242178

[46] Magdanz V Boryshpolets S Ridzewski C Eckel B Reinhardt K. The motility-based swim-up technique separates bull sperm based on differences in metabolic rates and tail length. PLoS ONE. (2019) 14:e0223576. 10.1371/journal.pone.0223576 31600297 PMC6786571

[47] Casao A Vega S Palacin I Perez-Pe R Lavina A Quintin FJ. Effects of melatonin implants during non-breeding season on sperm motility and reproductive parameters in rasa aragonesa rams. Reprod Domest Anim. (2010) 45:425–32. 10.1111/j.1439-0531.2008.01215.x 18954380

